# Zn-Doped MnCO_3_/CS Composite Photocatalyst for Visible-Light-Driven Decomposition of Organic Pollutants

**DOI:** 10.3390/molecules29051094

**Published:** 2024-02-29

**Authors:** Hui Liang, Yongxin Zhao, Tongjin Liu, Ruijuan Li, Rumei Li, Yuxiao Zhu, Feng Fang

**Affiliations:** 1 Institute of Plant Protection, Shandong Academy of Agricultural Sciences, Jinan 250100, China; huiliangchem@163.com (H.L.); tongjinliu2024@163.com (T.L.); ruijuanli@163.com (R.L.); rumeili0815@163.com (R.L.); sdgtzyx@163.com (Y.Z.); 2 College of Chemistry and Material Science, Shandong Agricultural University, Taian 271018, China; 15689525826@163.com

**Keywords:** visible-light-driven photocatalysis, carbon-based catalysts, organic pollutants

## Abstract

Zn-doped MnCO_3_/carbon sphere (Zn-doped MnCO_3_/CS) composites were synthesized using a simple hydrothermal procedure. Among various samples (ZM-50, ZM-05, and ZMC-0), the ternary Zn-doped MnCO_3_/CS (ZMC-2) catalyst demonstrated excellent visible light-induced photocatalytic activity. This improvement comes from the Zn addition and the conductive CS, which facilitate electron movement and charge transport. The catalyst exhibited efficient degradation of methylene blue (MB) over a wide pH range, achieving a removal efficiency of 99.6% under visible light. Radical trapping experiments suggested that •OH and •O_2_^−^ played essential roles in the mechanism of organic pollutant degradation. Moreover, the catalyst maintained good degradation performance after five cycles. This study offers valuable perspectives into the fabrication of carbon-based composites with promising photocatalytic activity.

## 1. Introduction

As industries grow, global concerns about environmental pollution are on the rise. One significant issue involves the release of organic dyes into wastewater during manufacturing and processing activities [1,2]. Among these organic dyes, methylene blue (MB) has been widely used in various industrial applications and medicine, which could be causing serious environmental and health problems. Various technologies such as physical adsorption, biodegradation, and advanced oxidation were developed for decomposing or degrading organic dyes in wastewater [3,4]. Compared with other traditional methods, photocatalysis technology has garnered more attention recently for eliminating various organic pollutants, because of its mild reaction conditions and ability to degrade thoroughly [3].

Heterogeneous photocatalysis based on inorganic semiconductors stands out as one of the most promising technologies for solving this issue due to its environmental friendliness, low energy consumption, and mild reaction conditions [3,4,5]. Semiconductor materials including TiO_2_, BiOBr, C_3_N_4_, CuO, ZnO, etc., have been extensively explored as photocatalysts for organic contaminant degradation [6,7,8,9,10]. It has been reported that highly porous TiO_2_ nanofibers produced in high humidity exhibit a MB degradation efficiency of 90% in 30 min, benefiting by its large surface area (128 m^2^/g) [6]. The introduction of carbon quantum dots could improve the band structure significantly. Zhong et al. prepared a carbon dot-doping C_3_N_4_/BiOBr heterostructure in order to enhance band regulation and electron transfer, resulting in 98.48% of rhodamine B (RhB) degradation within 15 min [7]. Xing et al. reported that CuO synthesized via the hydrothermal method displayed significant catalytic efficiencies in activating peroxymonosulfate for ciprofloxacin degradation. The presence of inorganic ions (Ca^2+^, Mg^2+^ and Cl^−^) had little effect on ciprofloxacin degradation and, under optimal conditions, ciprofloxacin would be completely degraded [9]. Sometimes, the selection of the components has led to miraculous synergies in removal of organic pollutants. As reported in Tian’s work [10], a ternary magnetic photocatalyst (ZnO@CoFe_2_O_4_@carbon nanotube) with excellent photocatalytic activity was synthesized for PMS activation under UVC light. The porous structure, separation of photogenerated electron-hole (e^–^-h^+^) pairs, and surface area are beneficial for enhancing the ternary photocatalyst activity on degradation of cefixime. Additionally, ZnO, CoFe_2_O_4_, and carbon nanotubes displayed high synergies during the degradation process; thus, 100% of cefixime was removed at 30 min. Despite this, many photocatalysts face challenges such as low solar utilization efficiency or a high recombination rate of photo-generated carriers [11,12]. Therefore, there is a widespread interest in exploring and preparing photocatalysts with broadened light absorption and enhanced carrier separation efficiency.

Manganese carbonate (MnCO_3_) is a well-known transition metal salt with high abundance, negligible toxicity, and a simple preparation procedure. However, MnCO_3_ has received limited attention in photocatalysis research owing to its low solar utilization efficiency and conductivity [13,14]. In the literature, the range of light absorption of the catalyst could be extended by introducing other metal ions [15]. In addition, it has been reported that doping with elements such as Zn^2+^, Bi^3+^, Fe^3+^, Ni^2+^, and Cu^2+^ can increase photocatalytic activity owing to enhanced electron exchange rates and a reduced band gap [16,17,18]. For instance, Zn doping enhances the charge separation and the utilization of visible light, and changes band gaps; therefore, porous Zn doped Zr^3+^-ZrO_2_ composite showed excellent photocatalytic activity for tetracycline degradation due to its narrow band gaps and broaden visible light [16]. Bhatia et al. reported that a TiO_2_ photocatalyst co-doped with Bi and Ni displayed higher photoactivity in ofloxacin degradation compared with TiO_2_ catalyst under visible light, and the concentration of Bi and Ni played an important role in photocatalytic activity. Moreover, metal ions act as trapping centers for photoinduced e^–^-h^+^ pairs, resulting in suppressed recombination of e^–^-h^+^ pairs, and the trapped charges are transferred to the surface of the semiconductor, thereby enhancing photocatalytic performance [17]. It has been reported that Cu- and Fe-doped TiO_2_ nanocatalysts reveal more lattice defects and a higher concentration of surface-bound hydroxyl groups/chemically adsorbed oxygen. Consequently, Cu-Fe-TiO_2_ composite has demonstrated significant photocatalytic activity, which can be attributed to its reduced band gap [18].

Recently, carbon-based materials have emerged as the preferred additive for enhancing the conductivity of catalysts. Previous studies have reported that carbon-based catalysts reduce the photo-generated e^–^-h^+^ recombination, extend excitation wavelengths, and increase the adsorption of reactants [19,20,21]. For instance, carbon dots/Ag nanoparticles/TiO_2_ nanocomposites demonstrated widened visible light absorption and reduced e^–^-h^+^ recombination rate. This effect was attributed to the synergism between the photoelectrical properties of the catalyst and carbon dots [22]. Acting as the electron transfer bridge, the incorporation of CS into the BiOBr/g-C_3_N_4_ composite improved the charge separation efficiency [23]. Studies have shown that Co-doped carbon aerogels, possessing large specific surface areas and pore volumes, efficiently eliminate organic contaminants by activating peroxymonosulfate species [24]. The synergy between the materials has been shown to improve the photocatalytic process. After the combination of Ag, TiO_2_, and carbon nanotubes, the photocatalytic activity was increased significantly because of the surface plasmon resonance effect of Ag [25]. Moreover, graphene, carbon foams, and other carbonaceous catalysts have been reported to significantly improve photocatalysts [26,27]. Among these carbon materials, CS stands out as a carrier due to its high surface area, excellent stability, low cost, and rich pattern of functional groups.

In this work, we report a straightforward hydrothermal synthesis of Zn-doped MnCO_3_/CS composite and explore its efficiency as a photocatalyst for the visible light decomposition of organic pollutants. The obtained composites were measured by XRD, SEM, TEM, and XPS, and the results reveal that the porous structure of the photocatalyst ensures a high contact area and a considerable number of active sites. Additionally, the integration of CS into the Zn^2+^/MnCO_3_ material enhances charge transport, resulting in outstanding photocatalytic degradation activity. In addition, the effect of various photocatalysts (tuned to the molar ratio of Zn/Mn and the amount of glucose, Table S1), initial pH, and recyclability of catalyst on MB degradation was explored. Based on the results of the free radical trapping experiment, a possible photocatalytic degradation mechanism has been proposed.

## 2. Results

**Figure 1:**
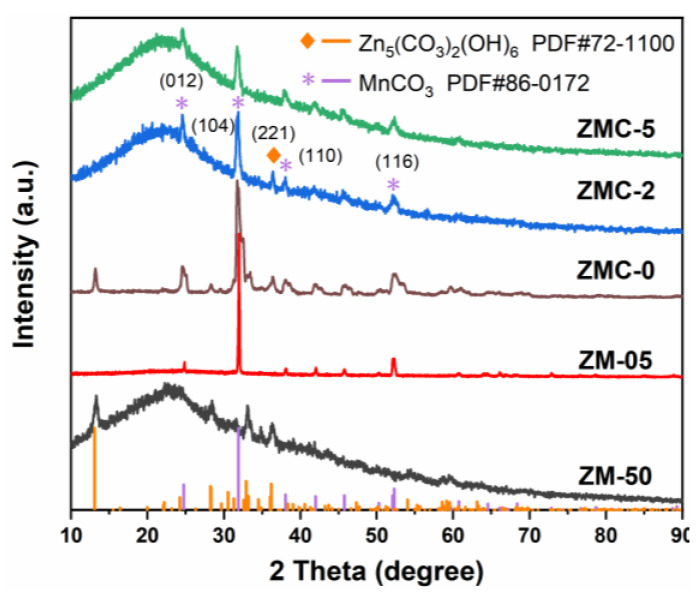
XRD spectra of the as-prepared materials.

Figure 1 presents the diffraction peaks of ZM-50, ZM-05, ZMC-0, ZMC-2, and ZMC-5 samples (preparation details of the five samples as shown in Table 1). The XRD patterns of ZM-50, ZM-05 (magnified in Figure S1), ZMC-2, and ZMC-5 exhibited characteristic diffraction peaks at 2*θ* = 23.2°, corresponding to the (002) plane of amorphous carbon [28]. In ZM-50, additional diffraction peaks correspond to the Zn_5_(CO_3_)_2_(OH)_6_ phase (JCPDS No. 72-1100) [29]. For ZM-05, aside from the carbon phase, peaks correspond to the MnCO_3_ phase (JCPDS No. 86-0172). Furthermore, typical peaks of Zn_5_(CO_3_)_2_(OH)_6_ and MnCO_3_ were observed in the diffraction patterns of ZMC-0, and in contrast, no peak of Zn_5_(CO_3_)_2_(OH)_6_ was observed in ZMC-5, which might be due to extensive coverage of CS. In ZMC-2, the diffraction peaks positioned at 36.2° corresponded to (221) crystal planes of Zn_5_(CO_3_)_2_(OH)_6_, and the peaks at 24.3°, 31.5°, 37.7°, and 51.9° matched well with the (012), (104), (110), and (116) planes of MnCO_3_, respectively, confirming the successful fabrication of the Zn-doped MnCO_3_/CS composite.

**Figure 2:**
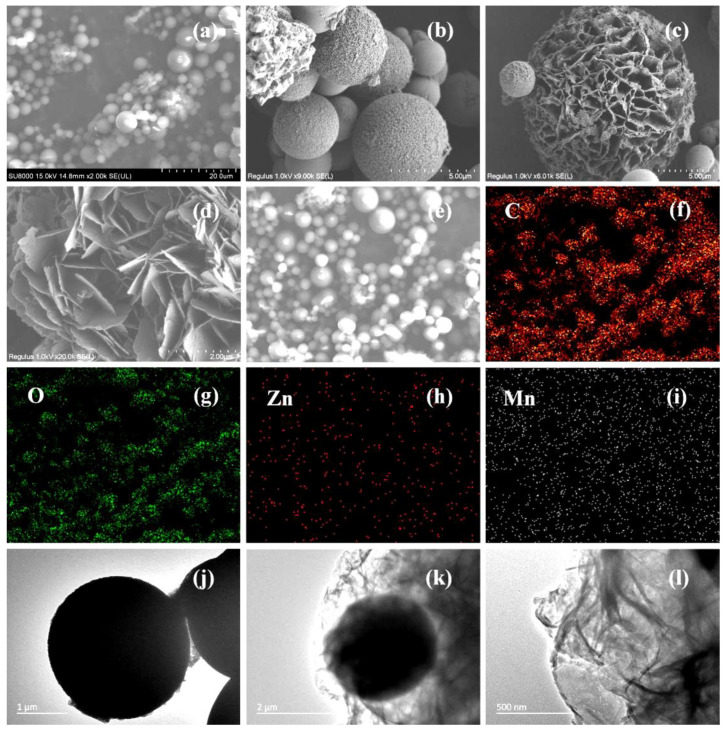
(**a**–**d**) SEM images, (**e**–**i**) elemental mapping, and (**j**–**l**) TEM images of ZMC-2 photocatalyst.

The morphology and elemental distribution of ZMC-2 were investigated using SEM and TEM. Figure 2a reveals that the sample was made of microspheres having diameters between 1 and 5 μm, featuring numerous smaller particles on their surface (Figure 2b). Moreover, a portion of the sample exhibited a hydrangea-like morphology, comprised of loosely arranged, hierarchical nanosheets with a thickness of ~20 nm (Figure 2c,d). Elemental mapping of the ZMC-2 catalyst (Figure 2e–i) reveals the evenly dispersed C, O, Zn, and Mn elements. TEM imaging (Figure 2j–l) further confirmed that the ZMC-2 photocatalyst comprised both solid microspheres and hydrangea-like structures.

**Figure 3:**
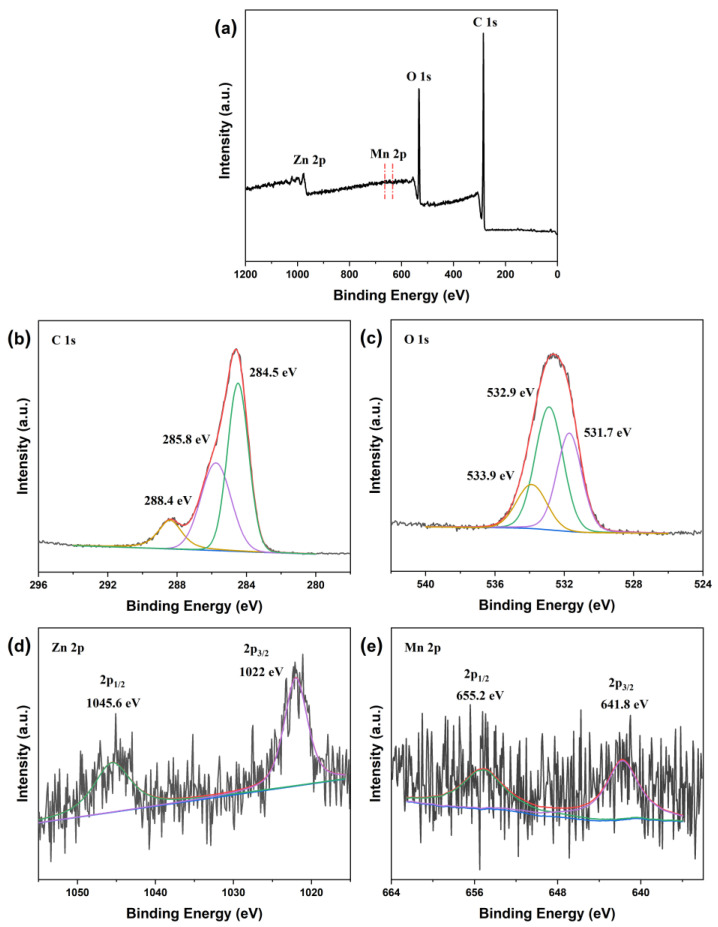
XPS data of the ZMC-2 photocatalyst: (**a**) survey spectrum, and spectra of (**b**) C 1s, (**c**) O 1s, (**d**) Zn 2p, and (**e**) Mn 2p levels.

To analyze the chemical composition of ZMC-2, XPS spectra were recorded, as depicted in Figure 3a. The characteristic maxima for C 1s, O 1s, Zn 2p, and Mn 2p electrons indicated the presence of C, O, Zn, and Mn in this material. Deconvolution of the C 1s peak (Figure 3b) revealed three Gaussians at 284.5, 285.8, and 288.4 eV that originated from C−C, C−O, and O−C=O groups, respectively [30,31,32]. In the O 1s XPS spectrum (Figure 3c), the main maximum at 531.7 eV corresponded to surface –OH groups, while two fitted peaks at 532.9 and 533.9 eV indicated the presence of carbonate ions [32,33].

The XPS data for the Zn 2p energy level (Figure 3d) indicated two maxima at binding energies of 1022 and 1045.6 eV. According to the literature, these peaks originate from the Zn 2p_3/2_ and Zn 2p_1/2_ levels of the Zn^2+^ ion [34,35]. Additionally, the Mn 2p XPS spectrum (Figure 3e) showed two characteristic maxima at 641.8 and 655.2 eV, associated with Mn 2p_3/2_ and Mn 2p_1/2_ states in the Mn^2+^ ion from MnCO_3_ [36,37]. These findings confirm the successful preparation of Zn-doped MnCO_3_/CS material.

**Figure 4:**
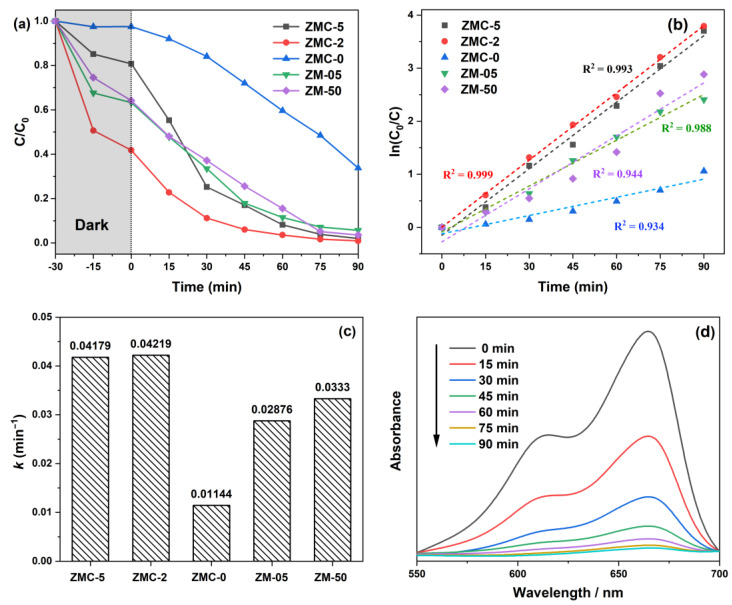
(**a**) Photocatalytic degradation curves of MB. (**b**) Fitted line for pseudo-first-order (PFO) kinetics, (**c**) the apparent rate constants (*k*), and (**d**) MB absorption spectra at different reaction times using the ZMC-2 photocatalyst.

The photocatalytic activity (PCA) of various composites prepared in this study was investigated by monitoring the visible-light-driven decomposition of the MB dye. The comparative performance of various photocatalysts is illustrated in Figure 4a. Adsorption equilibrium was achieved after 30 min, and the relative efficiencies decreased in the following order: ZMC-2 (99.06%) > ZMC-5 (98.01%) > ZM-50 (96.41%) > ZM-05 (94.29%) > ZMC-0 (66.20%). It is generally accepted that catalysts with porous structures exhibit enhanced performance in degrading organic pollutants [38]. In this context, carbon-based photocatalysts (ZM-50, ZM-05, ZMC-2, and ZMC-5) demonstrated higher PCA and the same pattern could be seen in Figure S2 (the removal efficiency values of MB over ZMC-1, ZMC-3, and ZMC-10 were 80.42%, 97.77%, and 97.20%, respectively, which were higher than that of ZMC-0), underscoring the significant role of carbon in the degradation mechanism.

Compared with ZM-50 and ZM-05, ZMC-2 showed the highest PCA, which suggests that the formation of ternary heterojunctions accelerates junctional electron flow, leading to efficient charge separation and additional redox reactions at the catalyst’s surface [39,40]. However, the PCA of the photocatalysts notably declined when the Zn/Mn molar ratio was changed to 1:4, 3:2, and 4:1 (Figure S2c,d. Information of the different photocatalysts varying the molar ratio of Zn/Mn and the quantity of C_6_H_12_O_6_ is shown in Table S1). In addition, the increase in the amount of glucose from 2 g to 3, 5, and 10 g resulted in a reduction in PCA from 99.06% to 97.77%, 98.01%, and 97.20%, respectively (Figure S2a). These results imply that excessive glucose leads to carbon agglomeration, damaging the ternary heterojunction structure and thereby inhibiting PCA [41].

Furthermore, the PCA data were fitted by the PFO kinetics model:

ln (*C*_0_/*C*) = *kt* (1)

where *C*_0_ and *C* represent the molarities of MB before and after photocatalytic treatment, respectively, and *k* is the corresponding apparent reaction rate constant. The excellent linearity of the plot presented in Figure 4b suggests that the photocatalytic degradation of MB followed PFO kinetics. The respective *k* values of ZMC-5, ZMC-2, ZMC-0, ZM-05, and ZM-50 were 0.04179, 0.04219, 0.01144, 0.02876, and 0.0333 min^−1^ (Figure 4c). In addition, as shown inset in Figure S2b,d, the *k* values of ZMC-1, ZMC-3, ZMC-10, ZM-14, ZM-32, and ZM-41 were 0.01204, 0.03839, 0.02915, 0.00811, 0.01591, and 0.01126 min^−1^. Obviously, ZMC-2 exhibited the highest photodegradation rate, approximately 3.7, 1.5, and 1.3 times greater than those of ZMC-0, ZM-05, and ZM-50 (3.5, 1.1, 1.4, 5.2, 2.7, 3.7 times higher than those of ZMC-1, ZMC-3, ZMC-10, ZM-14, ZM-32 and ZM-41), respectively. Moreover, the MB absorption peak gradually decreased with irradiation time, while the peak position showed no shift during degradation (Figure 4d). This consistent trend in the degradation process highlights the stability of the photocatalyst and the effective removal of MB from the solution over time.

**Figure 5:**
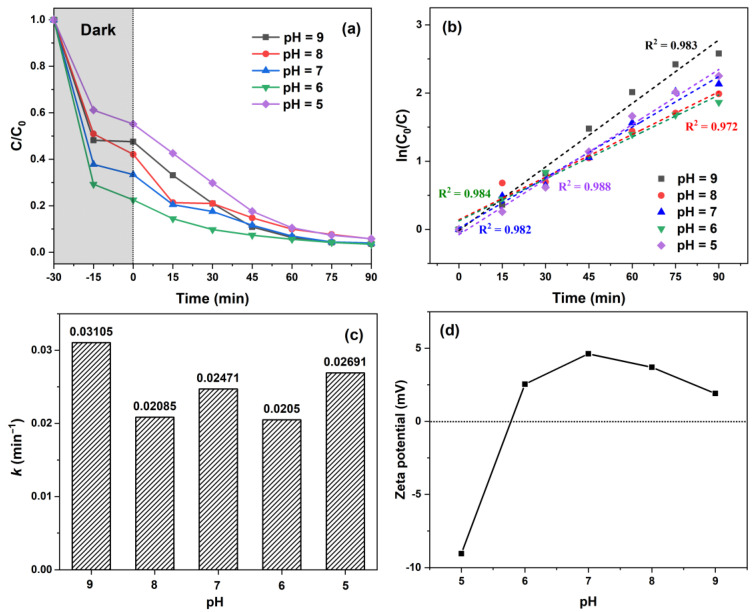
(**a**) Effect of pH values on the photocatalytic decomposition of MB; (**b**) kinetic profiles; (**c**) rate constant *k*; and (**d**) zeta potential of ZMC-2 at different pH values.

The pH of the solution has a large influence on the photocatalytic decomposition of organic pollutants [42,43,44]. Additionally, previous studies have shown the substantial impact of pH on the adsorption of small organic molecules onto catalyst surfaces [45,46]. In this experiment, the studied pH range was from 5 to 9, and the influence of initial pH on MB decomposition is illustrated in Figure 5. The adsorption performance of the catalyst increased gradually with decreasing pH (Figure 5a) and reached its peak at pH 6. However, a further decrease to pH 5 significantly diminished MB adsorption. 

The zeta potential of ZMC-2 is shown in Figure 5d, and the isoelectric point was 5.8, indicating that ZMC-2 was positively charged at pH > 5.8. If the electrostatic interaction is the only predominant adsorption mechanism, the removal of MB (cationic dyes) by ZMC-2 at pH 5 should be higher than that at the other pH values, because the ZMC-2 catalyst carries negative charge at this pH based on the zeta potential results. However, the highest MB removal was attained at pH 6, as observed in Figure 5a, indicating that other potential mechanisms played a role on the adsorption process, such as coordination interaction, π-π stacking, and H-bonding, etc. [19,47]. When the pH > 6, the adsorption efficiency of ZMC-2 decreased significantly, which could be attributed to the electrostatic repulsion between the positive charges of catalyst and the positively charged MB. In conclusion, the adsorption of MB on ZMC-2 was the result of the synergistic action of multiple mechanisms.

As depicted in Figure 5b,c, the order of the degradation rate constants was found to be pH 9 > 5 > 7 > 8 > 6. This indicates that the PCA exhibited irregular variations as the pH decreased. Notably, after 75 min of illumination, the removal efficiency of MB at various pH values became quite similar (94.2%, 96.5%, 96.0%, 94.2%, and 96.4%), suggesting that the pH of the starting solution had almost no impact on the long-term decomposition of MB, offering strong evidence for its practical application.

**Figure 6:**
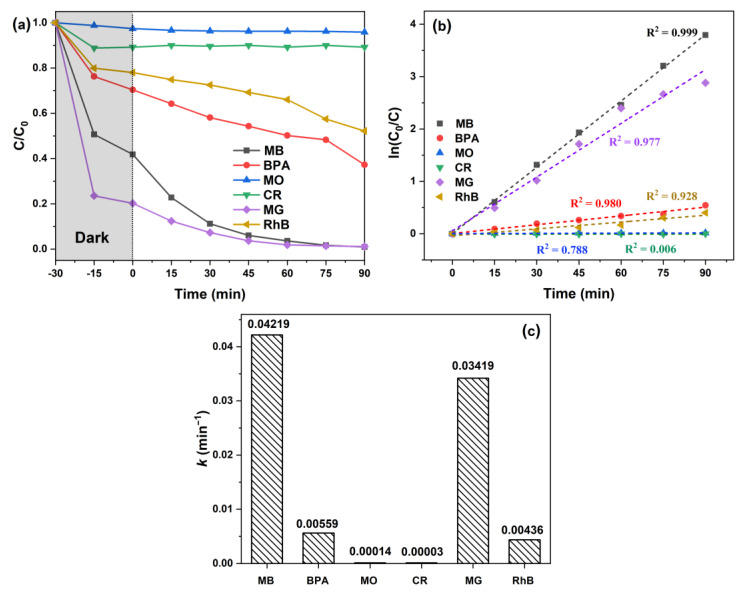
(**a**) Photocatalytic removal curves for different pollutants. (**b**) Data fitting into the PFO kinetics model and (**c**) the resulting *k* values. Reaction conditions: MB: 50 mg/L, BPA: 10 mg/L, MO: 20 mg/L, CR: 20 mg/L, MG: 50 mg/L, RhB: 20 mg/L, initial pH (unadjusted), catalyst dosage = 0.2 g/L.

To broaden the range of potential applications, we tested the photocatalytic activity (PCA) of the ZMC-2 catalyst on many organic pollutants other than MB under an identical experimental setup. Figure 6 summarizes the photocatalytic experiments and gives fitting lines for the removal of six structurally diverse organic pollutants. After 30 min of adsorption in the dark and 90 min of irradiation, the removal efficiencies for MB, BPA, MO, CR, MG, and RhB were found to be 99.06%, 62.76%, 4.14%, 10.77%, 98.86%, and 47.82%, respectively (Figure 6a). The data for each of these dyes aligned well into the first-order kinetic model, showing the corresponding *k* values were 0.04219, 0.00559, 0.00014, 0.00003, 0.03419, and 0.00436 min^−1^ (Figure 6c). 

The notable variations in removal efficiency across these different pollutants were expected, considering that photocatalytic degradation is highly influenced by the structural differences among organic pollutants [48,49,50]. This wide-ranging efficacy against various pollutants highlights the versatility and potential utility of the Zn-doped MnCO_3_/CS composite photocatalyst in treating diverse organic contaminants.

**Figure 7:**
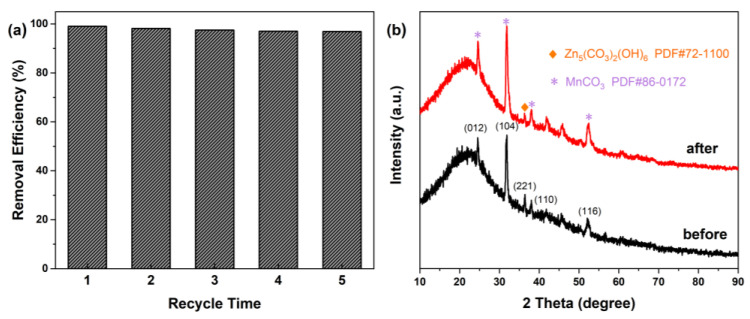
(**a**) Photodegradation of MB over ZMC-2 under visible light illumination and (**b**) XRD spectrum of ZMC-2 before and after 5 successive cycles.

Both the reproducibility and stability of a catalyst are essential parameters that limit its application [51]. The photoactivity of ZMC-2 showed only a slight reduction, from 99.06% to 96.88%, after undergoing five repeated runs (Figure 7a). This negligible decrease may be attributed to catalyst weight loss during the experiments. Moreover, Figure 7b illustrates that the crystal phase of the composites remained unchanged, indicating its exceptional photocatalytic stability.

**Figure 8:**
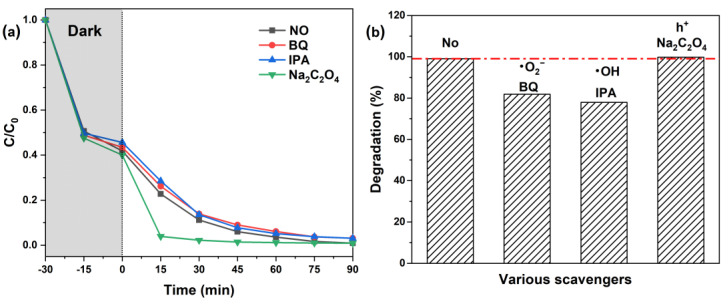
Free radical trapping experiments for visible-light MB photodegradation over ZMC-2. (**a**) Photocatalytic degradation curves, and (**b**) the removal rate.

To elucidate the mechanism of MB photodegradation over ZMC-2, we conducted radical trapping experiments to identify the main photoactive species. Benzoquinone (BQ), isopropanol (IPA), and sodium oxalate (Na_2_C_2_O_4_) were utilized as sacrificial agents for •O_2_^−^, •OH, and h^+^, respectively [52,53]. The photodegradation performance of ZMC-2 toward MB in the presence of various radical quenchers is displayed in Figure 8. When BQ and IPA were introduced, the initial MB removal rate (99.06%) decreased to 81.88% and 77.91%, respectively. These results suggest the participation of •OH and •O_2_^−^ radicals in the photodegradation mechanism of MB [54]. In addition, it was reported that reactive oxygen species (•OH and •O_2_^−^) played an important role not only in degradation process under visible light illumination but also in the dark (•OH and •O_2_^−^ species also can be produced under dark condition) [55,56]. However, in this work, the MB removal efficiency was slightly decreased (58.19% to 56.56%, 54.35%) in the dark in the presence of BQ and IPA (Figure S3), which indicated the primary reason for the abatement of MB was absorption rather than reactive oxygen species.

In addition, it is worth noting that the addition of Na_2_C_2_O_4_ triggered a fierce promotion of MB degradation, as shown in Figure 8a, and the removal rate of MB was significantly higher compared with other conditions within 60 min of irradiation. The possible reason is that the introduction of Na_2_C_2_O_4_ led to the capture of holes, thus improving the separation efficiency of electron-hole pairs, which is beneficial to the degradation of MB [57]. However, the promoting effect of Na_2_C_2_O_4_ decreased markedly after 75 min of irradiation, and the MB removal rate only increased to 99.80%, implying the dominant role of •OH and •O_2_^−^ in the degradation process.

**Figure 9:**
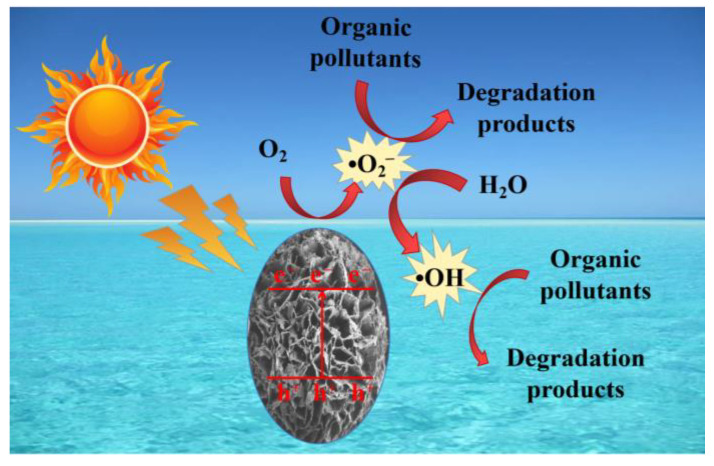
The plausible photocatalytic degradation mechanism of organic molecules by ZMC-2 visible-light photocatalyst.

Starting from the aforementioned discussion, we propose a plausible degradation mechanism for organic pollutants by ZMC-2 (Figure 9). During the adsorption stage, O_2_ and organic pollutants attach to the surface of ZMC-2 in the dark. Subsequently, visible light excitation drives e^−^ from the valence band (VB) to the conduction band (CB) of Zn-doped MnCO_3_, leaving abundant h^+^ in the VB. The good conductivity of the CS component of the photocatalyst facilitates the electron transfer to the surface, where they combine with the adsorbed O_2_ to yield •O_2_^−^. After that, a partial amount of •O_2_^−^ reacted with H_2_O to produce •OH. These radicals are identified as the primary species that participate in organic pollutant removal via the ZMC-2 catalyst.

## 3. Materials and Methods

### 3.1. Chemicals

Zinc acetate dihydrate, manganese chloride tetrahydrate, urea, glucose, ethanol, isopropyl alcohol, sodium oxalate, NaOH, and HCl were obtained from Sinopharm Chemical Reagent Co., Ltd. (Shanghai, China). Methyl orange, methylene blue, bisphenol A, and benzoquinone were acquired from Shanghai Macklin Biochemical Co., Ltd. (Shanghai, China). Congo red and malachite green were purchased from Aladdin Shanghai Co., Ltd. (Shanghai, China). 

All chemicals were of p.a. grade and were utilized without additional purification.

### 3.2. Synthesis

#### 3.2.1. Fabrication of Zn-Doped MnCO_3_

In a typical synthesis, 0.0878 g (0.4 mmol) of zinc acetate, 0.1188 g (0.6 mmol) of manganese chloride dihydrate, and 0.0901 g (1.5 mmol) of urea were added in 20 mL of deionized (DI) water and stirred for 30 min. The resulting solution was placed into a 25-mL Teflon-coated stainless steel autoclave and subjected to solvothermal synthesis at 180 °C for 4 h. After slowly cooling to RT, the resulting solid was centrifuged and subjected to three cycles of washing with DI water and EtOH. Following this, the solid was dried for 12 h at 60 °C and ground into powder for subsequent use.

#### 3.2.2. Fabrication of Zn-Doped MnCO_3_/CS Composites

For the synthesis, 0.1 g of Zn-doped MnCO_3_ and 2 g of glucose (C_6_H_12_O_6_) were dispersed in 40 mL of DI water. Next, the suspension was placed into a 50 mL Teflon-coated autoclave and allowed to react for 4 h at 180 °C. Subsequently, the products were collected once the system cooled down naturally to RT, and underwent multiple washes with DI water and absolute EtOH. The final product was gathered and dried at 60 °C for 1 day.

For comparative analysis, the samples were prepared by adjusting the quantity of C_6_H_12_O_6_ to 0, 2, and 5 g, denoted as ZMC-0, ZMC-2, and ZMC-5, respectively. Moreover, additional samples were synthesized according to the same procedure by varying the molar ratio of Zn(CH_3_COO)_2_/MnCl_2_ from 0.5:0 to 0:0.5, and labeled as ZM-50 and ZM-05. Further information on the experimental procedure is available in Table S1.

### 3.3. Characterization

The crystal structure was studied using powder XRD (Bruker D8, Bruker, Bremen, Germany) within a 2θ range from 10° to 80°. The morphology and the microstructure of the Zn-doped MnCO_3_/CS composite were examined by SEM (QUANTA250, FEI, SD, USA) and TEM (Tecnai G2 F20, FEI, SD, USA). The surface functional groups of the composites were analyzed by XPS (ESCALAB 250, Thermo Scientiffc, Waltham, MA, USA). UV-vis spectra were recorded on a UV-2540 spectrophotometer (Shimadzu, Tokyo, Japan). The zeta potential of the ZMC-2 at different pH values was measured using a Malvern Zetasizer Nano-ZS90 (Malvern Panalytical, Malvern, UK) instrument.

### 3.4. Photocatalytic Activity (PCA) Measurements

To assess the PCA of Zn-doped MnCO_3_/CS composites, we monitored the decomposition rate of methylene blue (MB). A visible light source (Xe lamp, 300 W, PerfectLight, Beijing, China) in a wavelength range above 420 nm was used, and the UV part of the spectrum was filtered out. In the standard experiment for monitoring the PCA, the solid catalyst (30 mg) was introduced into a 60 mL of 50 mg/L MB aqueous solution in a glass beaker. The resulting mixture was subjected to stirring in the dark for 30 min to equilibrate. Following this, the suspension underwent irradiation with white light (λ > 420 nm) under continuous stirring. During irradiation, 3 mL aliquots were taken at certain time steps, and the residual concentration of MB was monitored spectrophotometrically at 663 nm after removing solid particles through centrifugation. The solution’s pH was tuned with 0.1 M HCl and/or 0.1 M NaOH.

The degradation rate (*η*) of MB was analyzed by applying the equation *η* = *C*_t_/*C*_0_ × 100%, where *C*_0_ denotes the starting molarity of MB (before irradiation) and *C*_t_ is the molarity of MB at reaction time t.

## 4. Conclusions

In this work, we successfully prepared a Zn-doped MnCO_3_/CS material for the visible-light-driven removal of organic pollutants. Various characterization results revealed that the obtained composites were composed of microspheres and hydrangea-like morphology. Moreover, C, O, Zn, and Mn elements were uniformly dispersed throughout the materials. Among the prepared composites, ZMC-2 exhibited the most excellent performance in degradation of MB under visible light. The possible reason may be as follows. On the one hand, the inclusion of CS in the Zn-doped MnCO_3_ composite significantly enhanced charge transport, resulting in exceptional photocatalytic activity. On the other hand, the porous structure of the product not only increased the contact area but also increased the abundance of active sites, resulting in the photocatalytic removal efficiency of MB up to 99.6% within 120 min. Furthermore, this catalyst demonstrated stability over a wide pH range (5–9) and exhibited excellent recyclability, with an MB removal rate of 96.88% after four cycles. Quench experiments revealed that •O_2_^−^ and•OH were the major reactive species during the MB photocatalytic reaction process. These findings offer valuable insights for the complete removal of MB and structurally versatile pollutants from wastewater. Further research could explore the application of this composite in wastewater treatment and environmental remediation.

## Figures and Tables

**Table 1:** Preparation details of the main photocatalytic materials ^1^.

Samples	Molar Ratio of Zn/Mn	Amount of C_6_H_12_O_6_ (g)
ZMC-5	2:3	5
ZMC-2	2:3	2
ZMC-0	2:3	0
ZM-05	0:5	2
ZM-50	5:0	2

^1^ Further information on other catalysts is available in Table S1.

## Data Availability

Data are contained within the article and Supplementary Materials.

## Supplementary Materials

The following supporting information can be downloaded at: https://www.mdpi.com/article/10.3390/molecules29051094/s1, Table S1: The detailed experimental parameters; Figure S1: XRD pattern of as-prepared MnCO_3_/CS sample; Figure S2a,c: Photocatalytic activities of the as-prepared photocatalysts for the degradation of MB under simulated sunlight irradiation, respectively; Figure S2b,d: the corresponding reaction kinetics of the samples. Figure S3: Free radical trapping experiments for MB removal over ZMC-2 in dark.
