# Supplementary material for: Zn-Doped MnCO3/CS Composite Photocatalyst for Visible-Light-Driven Decomposition of Organic Pollutants
Source: Molecules. 2024 Feb 29;29(5):1094. doi: 10.3390/molecules29051094 (PMC10935289; doi:10.3390/molecules29051094)
Supplement: Supplementary file 1 [file molecules-29-01094-s001.zip › molecules-2848130-supplementary.pdf]

Supplementary Material

Title：Zn-doped $MnCO_3$/CS composite photocatalyst for visible-light-driven decomposition of organic pollutants

Table S1. The detailed experimental parameters.

Samples	Molar ratio of $Zn(CH_3COO)_2/MnCl_2$	Amount of $C_6H_{12}O_6$ (g)
ZMC-0	2:3	0
ZMC-1	2:3	1
ZMC-2 (ZM-23)	2:3	2
ZMC-3	2:3	3
ZMC-5	2:3	5
ZMC-10	2:3	10
ZM-50	5:0	2
ZM-41	4:1	2
ZM-32	3:2	2
ZM-14	1:4	2
ZM-05	0:5	2

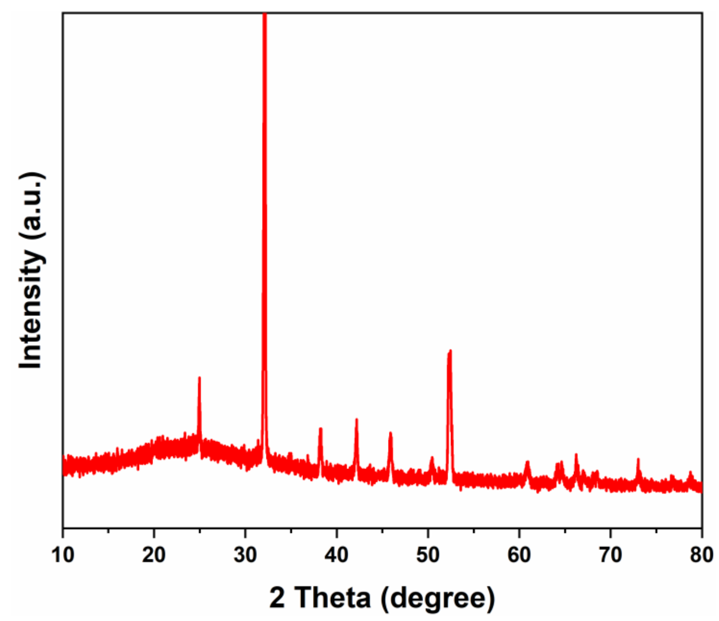

Figure. S1. XRD patterns of as-prepared $MnCO_3$/CS samples.

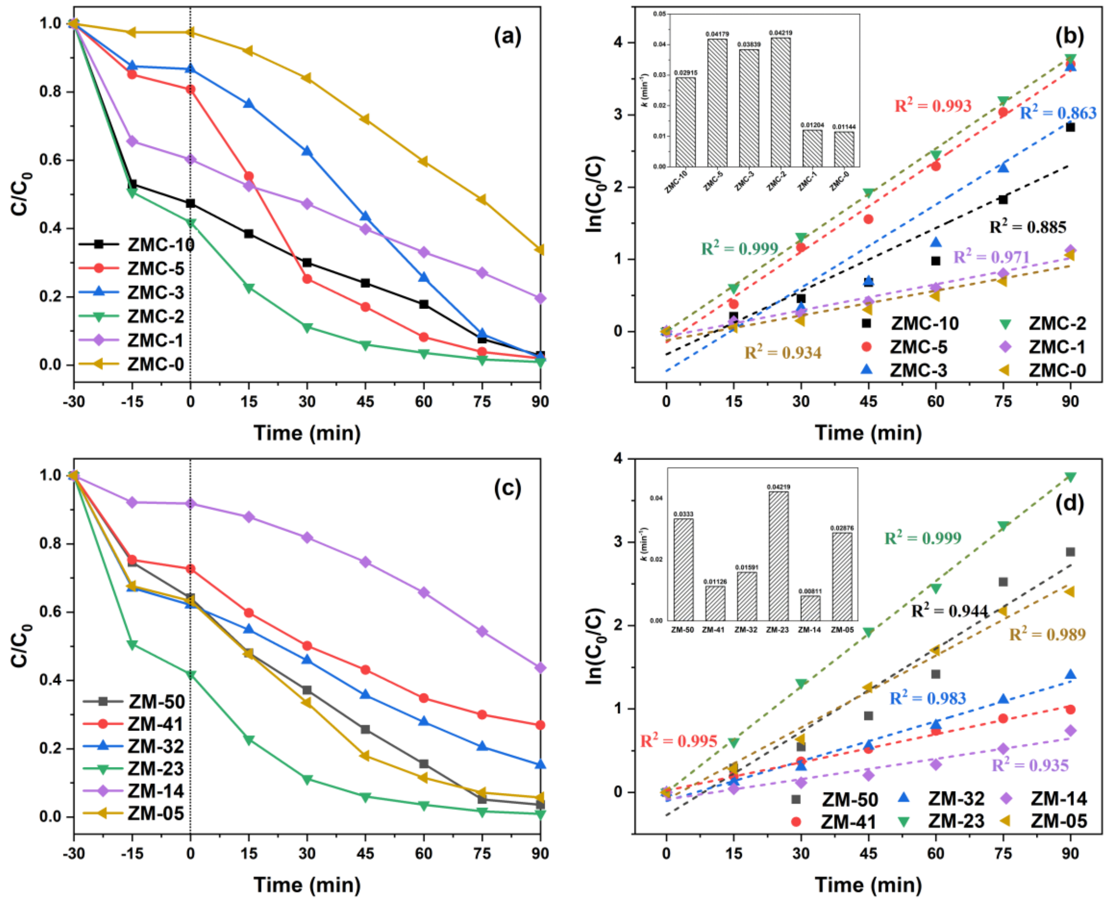

Figure. S2. (a, c) Photocatalytic activities of the as-prepared photocatalysts for the degradation of MB under simulated sunlight irradiation, respectively; (b, d) the corresponding reaction kinetics of the samples.

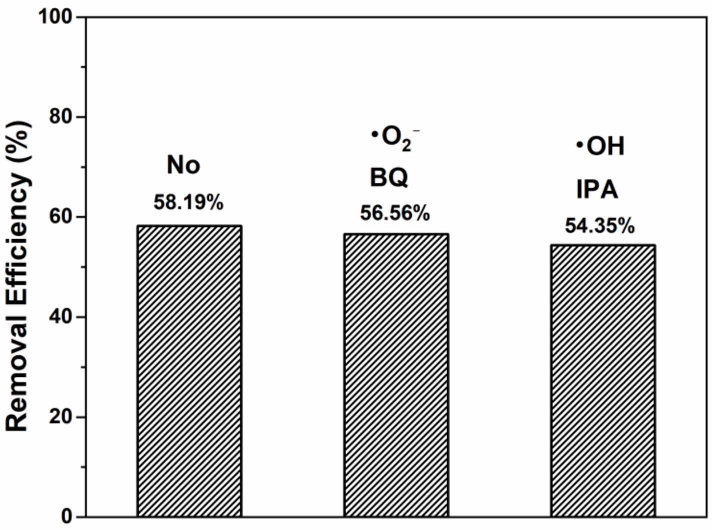

Figure. S3. Free radical trapping experiments for MB removal over ZMC-2 in dark.
